# Association between Neutrophil-to-Lymphocyte Ratio and Gut Microbiota in a Large Population: a Retrospective Cross-Sectional Study

**DOI:** 10.1038/s41598-018-34398-4

**Published:** 2018-10-30

**Authors:** Hee-Young Yoon, Han-Na Kim, Su Hwan Lee, Soo Jung Kim, Yoosoo Chang, Seungho Ryu, Hocheol Shin, Hyung-Lae Kim, Jin Hwa Lee

**Affiliations:** 1 Division of Pulmonary and Critical Care Medicine, Department of Internal Medicine, College of Medicine, Ewha Womans University, Seoul, Republic of Korea; 2 Medical Research Institute, Kangbuk Samsung Hospital, Sungkyunkwan University, School of Medicine, Seoul, South Korea; 3 Center for Cohort Studies, Total Healthcare Center, Kangbuk Samsung Hospital, Sungkyunkwan University, School of Medicine, Seoul, South Korea; 4 Department of Occupational and Environmental Medicine, Kangbuk Samsung Hospital, Sungkyunkwan University, School of Medicine, Seoul, South Korea; 5 Department of Family Medicine, Kangbuk Samsung Hospital, Sungkyunkwan University School of Medicine, Seoul, South Korea; 6 Department of Biochemistry, College of Medicine, Ewha Womans University, Seoul, Republic of Korea

## Abstract

Gut microbiota and blood neutrophil-to-lymphocyte ratio (NLR) are associated with systemic inflammation; however, data on the association between gut microbiota and NLR are lacking. We investigated the association between gut microbiota and NLR. A total of 1,309 subjects who had available data on NLR and 16 S rRNA sequencing of gut microbiota were included in this study. They were grouped according to NLR quartile (Q) as follows: lower Q (n = 328, <25% of NLR range), middle 2Q (n = 653, ≥25% to <75%) and upper Q (n = 328, ≥75%). The diversity and composition of the human gut microbiota in the groups were calculated. The phylogenetic diversity of gut microbiota in the lower group was significantly higher than in the middle 2Q group (*P* = 0.040). The beta-diversity was significantly different among the three groups (*P* = 0.043), between the lower and middle 2Q groups (*P* = 0.029), and between the lower and upper groups (*P* = 0.026). *Bacteroides eggerthii* showed a positive correlation with NLR (q = 0.015). The diversity and composition of the gut microbiome were different between the NLR groups. Particularly, patients with a lower NLR had a greater diversity of gut microbiota.

## Introduction

The human intestinal tract contains a varied and complex microbiotic environment that consists of more than 1,000 different species whose DNA encode the proteins for 3 million microbiomes^1^. Because the composition of gut microbiota is affected by multiple factors, such as host genetics, diet, geographic location, environment, early microbial exposure, and use of medication^2–5^, the diversity and abundance of the gut microbiota greatly differ between healthy individuals. However, functional metabolic pathways remain relatively consistent despite variations in the microbiota^6^. The crucial role of gut microbiota consists of its association with development and maintenance of the human immune system via metabolism^7,8^. Recently, several studies have reported that the gut microbiota influences the pathogenesis of immune-mediated inflammatory diseases such as inflammatory bowel disease, multiple sclerosis, rheumatoid arthritis, and ankylosing spondylitis^7,9^.

Neutrophil-to-lymphocyte ratio (NLR), which is calculated by dividing the absolute count of neutrophils by the number of lymphocytes in the complete blood count, is a simply-measured and reproducible biomarker used to evaluate systemic inflammation^10,11^. NLR is not only highly correlated with the prognosis of cancer patients, but it is also correlated with the prognosis of multiple diseases including inflammatory disease, cardiovascular disease, and tuberculosis^12–19^. Patients with an elevated NLR show a relatively decreased number of lymphocytes and an increased number of neutrophils, suggesting that cell-mediated immunity is impaired and systemic inflammation is increased in inflammatory processes^13,16,20^. Although Forget *et al*. reported that the normal value of NLR ranged from 0.78 to 3.53 in the healthy population^21^, the range of normal NLR is still controversial due to limited evidence that subjects with a normal NLR range have similar immunity and inflammatory statuses.

We hypothesized that the composition and diversity of human gut microbiota are different depending on the range of NLR since both gut microbiota and NLR are affected by systemic inflammation and immunity in healthy individuals. The purpose of our study is to investigate the association between NLR and gut microbiome in a large-scale population.

## Results

### Baseline characteristics of the subjects

**Figure 1:**
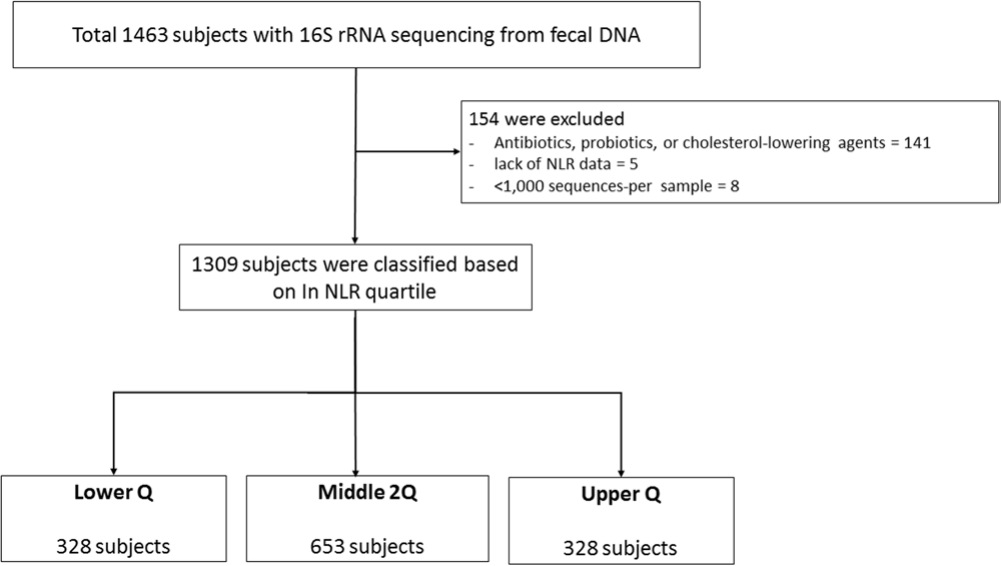
Enrollment of subjects. NLR, neutrophil-lymphocyte-ratio; RNA, RiboNucleic Acid; DNA, DeoxyriboNucleic Acid; Lower Q, <25% of NLR range; Middle 2Q, ≥25% to <75% of NLR range; Upper Q ≥ 75% of NLR range.

Of the total 1,309 subjects (mean age, 45.7 years; males, 62.3%; mean NLR, 1.67), 328 (25.1%), 653 (49.9%), and 328 (25.1%) were classified as the lower Q, middle 2Q, and upper Q groups, respectively (Table 1 and Fig. 1). The values of the first and third NLR quartiles were 1.25 and 1.98, respectively. No significant differences in demographics were observed between the three groups, except BMI and smoking status. In laboratory findings, the mean NLR was 1.1, 1.6, and 2.6 in the lower Q, middle 2Q, and upper Q groups, respectively. The total white blood cell count (5.3 10^3^/mm^3^ [lower Q] *vs*. 5.7 10^3^/mm^3^ [middle 2Q] *vs*. 6.5 10^3^/mm^3^ [upper Q], *P* < 0.001) and the proportion of neutrophils were significantly higher in the upper Q group than in the lower Q and middle 2Q groups (45.1% *vs*. 55.2% *vs*. 65.0%, *P* < 0.001), whereas the proportions of lymphocytes (44.7% *vs*. 35.3% *vs*. 26.3%, *P* < 0.001), eosinophils (3.0% vs. 2.6% vs. 2.2%, *P* < 0.001), basophils (0.5% vs. 0.5% vs. 0.4%, *P* < 0.001), and monocytes (6.6% vs. 6.4% vs. 6.1%, *P* < 0.001) were higher in the lower Q group compared with upper Q and middle 2Q groups. C-reactive protein (CRP) in the upper group was also significantly higher than in the other groups (0.09 mg/dL *vs*. 0.09 mg/dL *vs*. 0.10 mg/dL, *P* = 0.002). Differences in comorbidities and nutritional intake were not identified between the three groups (Supplement Table S1 and S2).

**Table 1:** Baseline characteristics according to neutrophil-to-lymphocyte-ratio quartile.

Variables	Lower Q	Middle 2Q	Upper Q	P-value
No.	328	653	328	
Age, years	46.4 (8.9)	45.3 (8.8)	45.6 (9.2)	0.173
Male	214 (65.2)	403 (61.7)	195 (59.5)	0.302
Body mass index, kg/m^2^	23.7 (2.9)	23.8 (3.2)	23.2 (3.2)	0.012
Smoking status				0.024
Never	168 (56.0)	363 (59.2)	189 (61.2)	
Former	69 (23.0)	133 (21.7)	83 (26.9)	
Current	63 (21.0)	117 (19.1)	37 (12.0)	
Smoking amount, pyrs	6.0 (9.7)	6.3 (11.0)	5.4 (9.7)	0.426
NLR	1.1 (0.2)	1.6 (0.2)	2.6 (0.8)	<0.001
White blood cell, 10^3^/mm^3^	5.3 (1.3)	5.7 (1.3)	6.5 (1.7)	<0.001
Neutrophil, %	45.1 (4.6)	55.2 (3.4)	65.0 (4.4)	<0.001
Lymphocyte, %	44.7 (4.4)	35.3 (2.9)	26.3 (3.6)	<0.001
Eosinophil, %	3.0 (2.2)	2.6 (2.1)	2.2 (1.8)	<0.001
Basophil, %	0.5 (0.3)	0.5 (0.3)	0.4 (0.2)	<0.001
Monocyte, %	6.6 (1.6)	6.4 (1.7)	6.1 (1.5)	<0.001
Hemoglobin, g/dL	14.3 (1.4)	14.3 (1.5)	14.2 (1.6)	0.266
Platelet, 10^3^/mm^3^	243.5 (51.8)	247.1 (60.8)	252.0 (58.3)	0.169
C-reactive protein, mg/dL	0.09 (0.19)	0.09 (0.13)	0.10 (0.17)	0.002

Data are presented as mean (standard deviation) or number (%).

NLR, neutrophil-lymphocyte-ratio; Lower Q, <25% of NLR range; Middle 2Q, ≥25% to <75% of NLR range; Upper Q ≥ 75% of NLR range; pyrs, pack-years.

We made additional comparisons between the lower Q and higher 3Q groups (Supplement Table S3); no significant differences in demographics were identified. In the laboratory findings, the trends were similar to those of the three groups, except there was no statistical difference in the level of CRP (0.09 [lower Q] vs. 0.11 [higher 3Q], *P* = 0.222)

### Comparison of alpha diversity within and between the NLR groups

**Figure 2:**
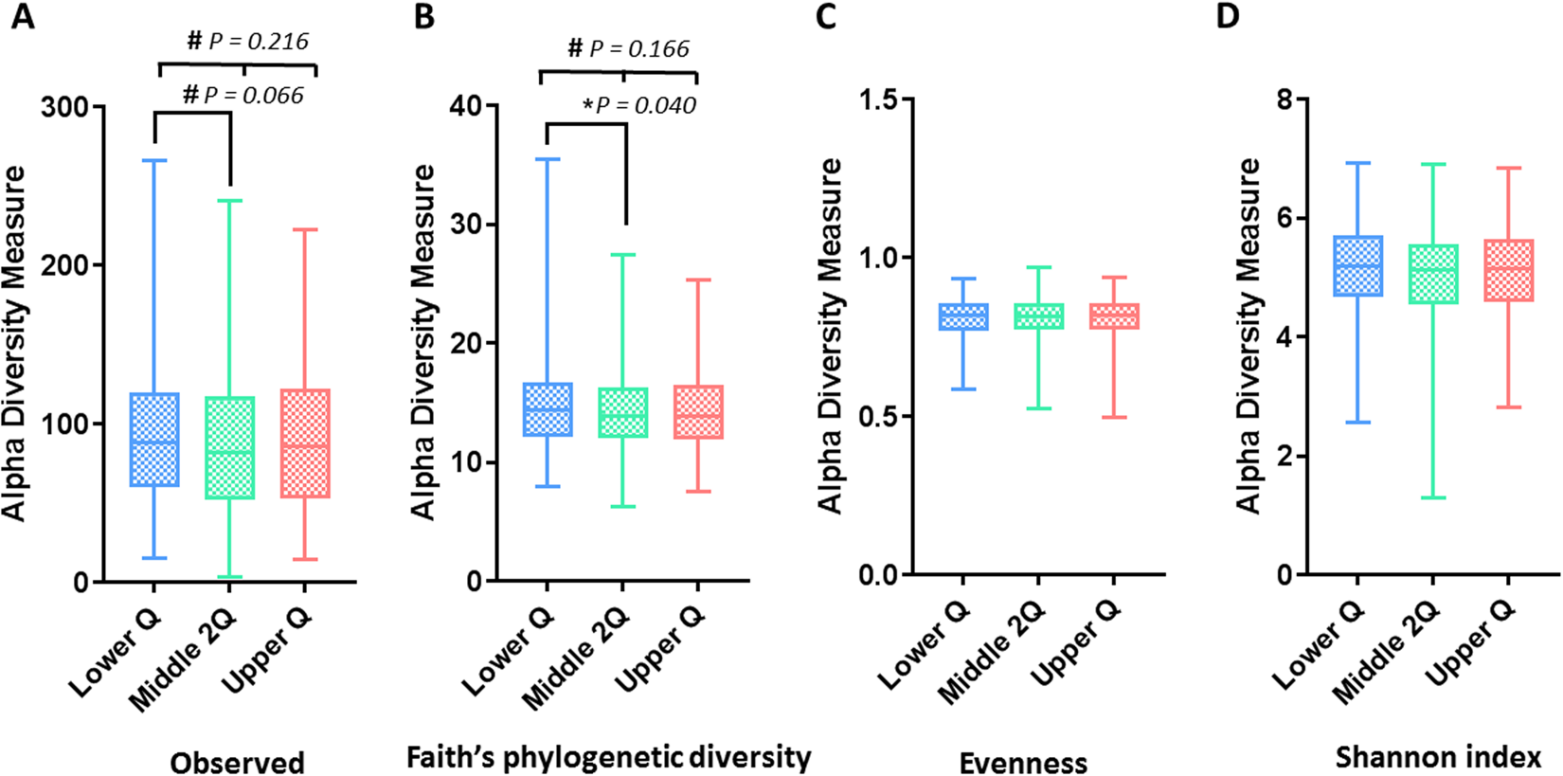
Box plots of alpha-diversity indices comparing neutrophil-to-lymphocyte-ratio groups. (**A**) Observed OTU, (**B**) phylogenetic diversity, (**C**) Pielou evenness, and (**D**) Shannon’s index. OTU, operational taxonomic unit; Lower Q, < 25% of NLR range; Middle 2Q, ≥25% to <75% of NLR range; Upper Q ≥ 75% of NLR range. *P < 0.05; ^#^*P* < 0.25.

The alpha diversity of gut microbial taxa between the lower Q, middle 2Q, and upper Q groups did not show statistically significant differences in observed OTU (93.9 ± 45.3 [lower Q] vs. 88.3 ± 45.5 [middle 2Q] vs. 90.4 ± 44.0 [Upper Q], *P* = 0.216), PD (14.8 ± 3.7 vs. 14.3 ± 3.5 vs. 14.5 ± 3.5, *P* = 0.166), evenness (0.81 ± 0.07 vs. 0.81 ± 0.07 vs. 0.81 ± 0.0, *P* = 0.986), and Shannon’s index (5.14 ± 0.76 vs. 5.06 ± 0.75 vs. 5.08 ± 0.75, *P* = 0.313) (Fig. 2). However, PD was significantly higher in the lower Q group than in the middle 2Q group (*P* = 0.040) in the post hoc test. These results indicate that richness, including phylogenetic diversity, was different among the NLR groups, but evenness was not.

In the comparison between the lower Q and higher 3Q groups, the lower Q group showed marginal trends of significance of alpha diversity in PD (14.8 ± 3.7 [lower Q] vs. 14.3 ± 3.4 [higher 3Q], *P* = 0.055) and observed OTU (93.9 ± 45.3 vs. 89.0 ± 43.0, *P* = 0.090) compared with the higher 3Q group (Supplement Figure S1).

### Comparison of beta diversity within and between the NLR groups

**Figure 3:**
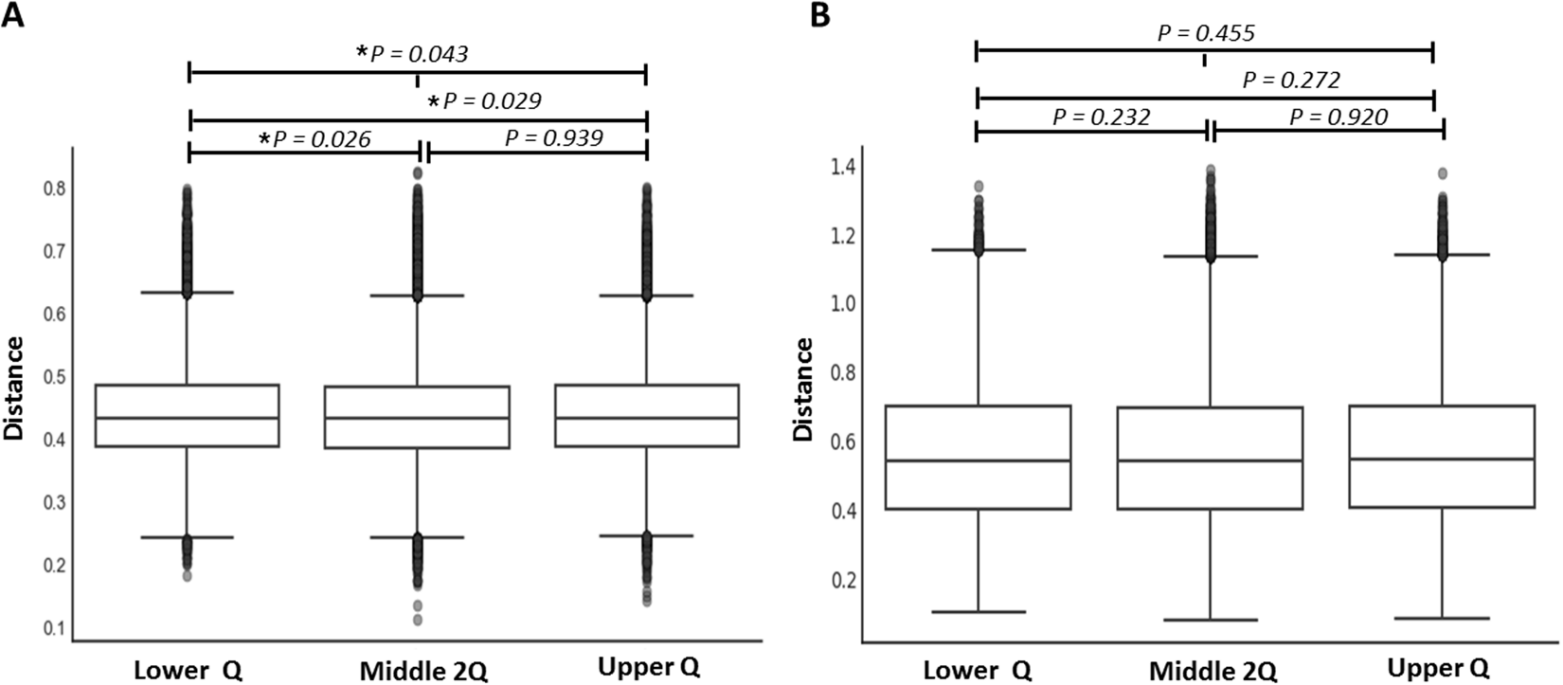
Comparison distance of beta diversity between neutrophil-to-lymphocyte-ratio groups. (**A**) Distance from the lower group using unweighted UniFrac and (**B**) Distance from the lower group using weighted UniFrac. Lower Q, <25% of NLR range; Middle 2Q, ≥25% to <75% of NLR range; Upper Q ≥ 75% of NLR range. *P < 0.05.

Unweighted UniFrac-based diversity was significantly different among the lower Q, middle 2Q, and upper Q groups (*P* = 0.043); between the lower Q and upper Q groups (*P* = 0.029); and between the lower Q and middle 2Q groups (*P* = 0.026; Fig. 3A). However, the unweighted UniFrac-based diversity was not significantly different between the middle 2Q and upper Q groups (*P* = 0.939). In weighted UniFrac-based beta diversity, no significant differences were revealed among the three groups *(P* = 0.455) or within each of the two groups (Fig. 3B).

There was a significant difference in unweighted UniFrac-based diversity between the lower Q and higher 3Q groups, but the differences were very small (*P* = 0.013; Supplement Figure S2). Results of weighted UniFrac-based beta diversity were not significantly different between the lower Q and higher 3Q groups (*P* = 0.175; Supplement Figure S2B).

### Associations between gut microbiota composition and NLR and total WBC count using quantitative analysis

**Figure 4:**
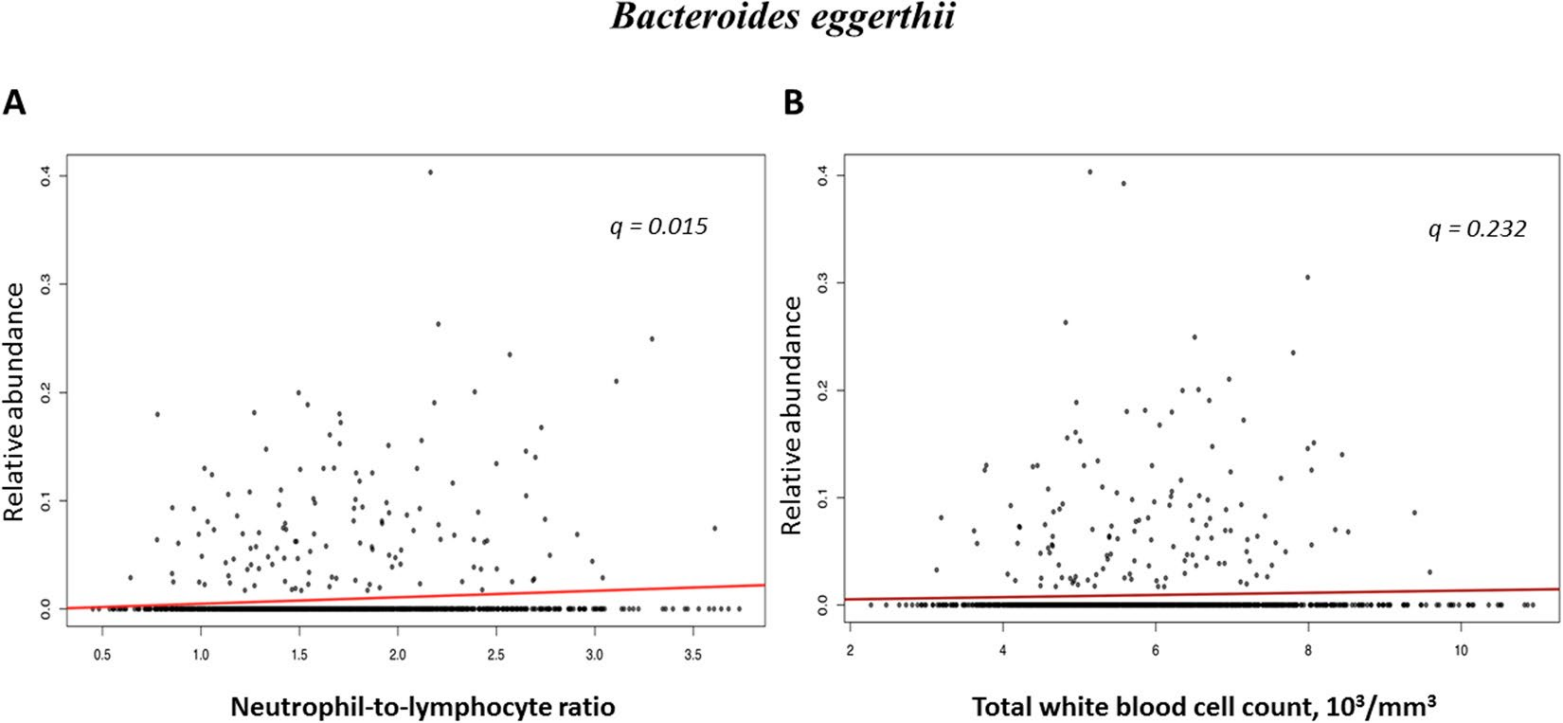
Correlations of *Bacteroides eggerthii* with neutrophil to lymphocyte ratio and white blood cell count. (**A**) Correlation with neutrophil to lymphocyte ratio and (**B**) Correlation with white blood cell count.

In the analysis of correlation between gut microbial taxa abundance and NLR after adjusting for age, sex, smoking status, and BMI, one species (*Bacteroides eggerthii* which belongs to the family Bacteroidaceae) was positively correlated with NLR (CE, 0.00605; *P* = 0.001; q = 0.015; Table 2 and Fig. 4A) and showed a positive association trend with total WBC count (CE, 0.00154; *P* = 0.049; q = 0.232; Fig. 4B). The genera *Dialister* and *Prevotella stercorea* had trends of a positive (CE, 0.01059; *P* = 0.024; q = 0.132) and negative correlation (CE, −0.00864; *P* = 0.037; q = 0.190) with NLR, respectively (Table 2).

**Table 2:** Correlations of identified taxa with neutrophil-lymphocyte-ratio using MaAsLin analysis.

Order	Family	Genus	Specie	N not to zero (%)	CE	P-value*	q-value*
Clostridiales	Veillonellaceae	*Dialister*		770 (58.8)	0.01059	0.024	0.132
Bacteroidales	Bacteroidaceae	*Bacteroides*	*eggerthii*	137 (10.5)	0.00605	0.001	0.015
Bacteroidales	Prevotellaceae	*Prevotella*	*stercorea*	449 (34.3)	−0.00864	0.037	0.190

CE, coefficient.

*Adjusted for age, sex, body mass index, and smoking status.

The regression CE represents the rate of change in abundance of taxa per 1 NLR.

### Comparison of gut microbiota composition between the NLR groups using qualitative analysis

**Figure 5:**
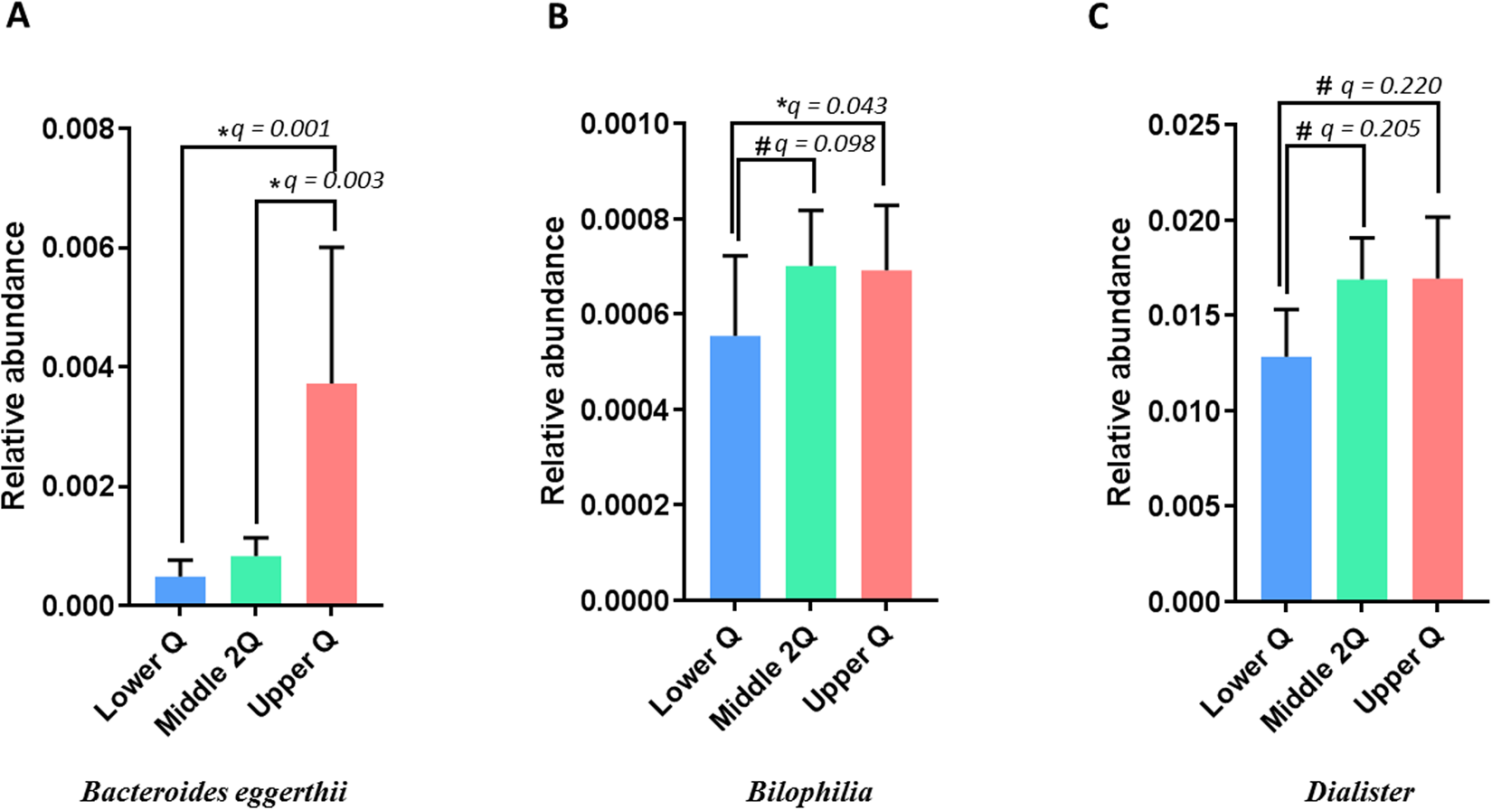
Comparison of relative abundance between neutrophil-lymphocyte-ratio groups. (**A**) *Bacteroides eggerthii*, (**B**) genus *Bilophila*, and (**C**) genus *Dialister*. Bars and error bars show the mean ± 95% confidence interval. Lower Q, < 25% of NLR range; Middle 2Q, ≥25% to <75% of NLR range; Upper Q ≥ 75% of NLR range. *q < 0.05; ^#^q < 0.25.

The relative abundance of gut microbiota was compared between groups using MaAsLine. The upper Q group had a significantly higher proportion of *Bacteroides eggerthii* compared with the lower Q (CE, 0.0119; *P* < 0.001; q = 0.001) and middle 2Q groups (CE, 0.00978; *P* < 0.001; q = 0.003; Fig. 5A). This significant trend of *Bacteroides eggerthii* held in the quantitative analysis. The relative abundance of the genus *Bilophilia* was significantly lower in the lower Q group than in the upper Q group (CE, 0.0045; *P* = 0.004; q = 0.043) and showed a marginal trend toward decrease compared with the middle 2Q group (CE, 0.0034; *P* = 0.014; q = 0.098; Fig. 5B). The abundance of *Dialister* organisms at the genus level in the lower Q group also showed a decreasing trend compared with the upper Q (CE, 0.0148; *P* = 0.045; q = 0.220) and middle 2Q groups (CE, 0.0129; *P* = 0.043; q = 0.205; Fig. 5C).

In the comparison between the lower Q and higher 3Q groups, the relative abundance of the genus *Bilophila* in the lower Q group was significantly lower than that in the higher 3Q group (CE, 0.0038; *P* = 0.004; q = 0.036; Supplement Figure S3A). The relative abundance of the genera *Dialister* (CE, 0.0135; *P* = 0.024; q = 0.143; Supplement Figure S3C) and *Bacteroides eggerthii* (CE, 0.0054; *P* = 0.027; q = 0.145; Supplement Figure S3D) had decreasing trends in the lower Q group than in the higher 3Q group, while abundance of *Prevotella stercorea* (CE, −0.0124; *P* = 0.019; q = 0.119; Supplement Figure S3B), the genus *Phascolarctobacterium* (CE, −0.0111; *P* = 0.035; q = 0.178; Supplement Figure S3E), and the genus *Lachnospira* (CE, −0.0081; *P* = 0.048; q = 0.229; Supplement Figure S3F) in the lower Q group showed trends toward increase compared with those in the higher 3Q group.

## Discussion

This is the first population-based study that identified correlations between NLR and gut microbiota in a population with a relatively normal NLR range. The gut microbiome in the lower Q NLR group showed higher richness in alpha diversity analysis. We also found differences in beta diversity among the NLR groups, particularly among the lower Q and other upper groups. The relative abundance of *Bacteroides eggerthii* was positively correlated with the value of NLR and was significantly elevated in the upper Q group compared with the lower Q and middle 2Q groups. When comparing the lower Q and higher 3Q groups, the genus *Bilophila* was less abundant in the lower Q group than in the higher 3Q group.

NLR can be used as a prognostic marker in multiple diseases^12–14,17–19,22,23^. The value of NLR ranges from 0.78 to 3.53 in the healthy adult population^21^. Although there is controversy on the optimal NLR cut-off values, numerous studies have reported that the NLR cut-off value for mortality in specific diseases is between 3 and 5^17,23–25^. The cut-off value of NLR for disease severity and activity in inflammatory bowel disease (IBD) is slightly lower compared with other medical conditions, ranging from 2 to 3^26–28^. Our study demonstrated a subtle difference in beta diversity between the lower and upper 3Q groups. Considering that we divided the NLR groups based on a cut-off of 1.25, which was considered a normal value in previous studies, the composition of the microbiota might be different depending on the NLR, even within the normal range. In addition, these subtle differences might be due to the healthy population.

NLR and CRP can be used as markers of systemic inflammation in various medical settings. CRP is an acute-phase protein produced in the hepatocytes, exposed to the inflammatory cytokines and tumor necrosis factor. CRP is rapidly increasing in response to inflammation and used as a marker reflecting prognosis and disease severity in various inflammatory situations including infection, trauma, and tumor^29^; however, it is difficult to interpret because the cause of inflammation is not well discriminated. On the other hand, because NLR represents the function of lymphocyte, it can more accurately reflect both the inflammatory response and the immune state^30,31^. Therefore, the NLR can most sensitively reflect the microbiome changes caused by external factors, such as immune systems or inflammatory responses.

The alpha diversity indices were also higher in the lower Q group than in the upper 3Q group. These findings suggest that, even within the normal rage of NRL, alpha diversity (particularly, the richness) of gut microbiota decreases as NLR increases. We also found that the abundance of several gut microorganisms had a linear correlation with NLR in the healthy population. This is consistent with previous reports that the activity scores of several diseases have a linear correlation with NLR^32,33^. In conclusion, even within the normal range, the lower is the NLR, the lower is the risk for inflammatory disease, which is similar to the findings that the lower is the blood glucose or blood pressure, the lower is the risk of diabetes mellitus or cardiovascular-related mortality, respectively^34,35^.

*B. eggerthii* is a less commonly isolated species of *Bacteroide*, which is a genus of Gram-negative, anaerobic, non-spore-forming bacteria that is part of the normal human gastrointestinal flora^36–38^. Using *in vivo* mouse models, Dziarski *et al*. indicated that an abundance of *B. eggerthii* is associated with dextran sulfate sodium-induced colitis^39^. In addition, several reports have suggested that changes in abundance of *Bacteroide* are observed in subjects with IBD, including patients with Crohn’s disease and ulcerative colitis^40–42^. On the other hand, NLR is a biomarker of disease activity and prognosis in patients with IBD^14,26,27^. Considering previous reports indicating the relationships between colitis and NLR and between colitis and *B. eggerthii*, our study showed a significant association between NLR and *B. eggerthii*. Therefore, there is likely a link among NLR, *B. eggerthii*, and intestinal inflammation.

*Biophilia spp*. are Gram-negative, anaerobic, urease-positive, bile-resistant bacteria associated with an animal-based protein and high-saturated fat diet, and they promote T helper type 1 (Th1)-mediated immunity^43^. The most frequently isolated species of *Biophilia* is *B. wadsworthia*, and although it is found in < 0.1% of the normal human gastrointestinal flora, it is found in abundance in infectious conditions such as colitis, perforation, and abscesses^43,44^. Devkota *et al*. demonstrated that *B. wadsworthia* induced colitis in IBD-prone *interleukin (IL)-10* mice by activating Th1-mediated colonic inflammation^45^. We identified a lower abundance of *Biophilia* in the lower Q NLR group than in the higher 3Q group. Considering that NLR is a marker of prognosis and severity of IBD, an elevated abundance of *B. eggerthii* and genus *Biophilia* might be associated with the status of bowel inflammation, and NLR could reflect these microbiota changes, even though our subjects were relatively healthy.

Our results suggest that several organisms of the gut microbiota differ in abundance between the lower Q and higher Q3 groups (q-value < 0.2). The genus *Dialister*, which showed an increasing trend of abundance in the higher Q3 NLR group, has been associated with irritable bowel syndrome^46,47^. Organisms of the genus *Prevotella*, considered to have a beneficial role due to its association with a plant-rich diet^48^, showed a decreased quantity in the higher Q3 NLR group. These results suggest that NLR decreases as the gut microbiota changes within a beneficial environment. Furthermore, since NLR is related to the prognosis of various medical conditions^12–14,17–19,22,23^, it is suggested that small changes can reflect the early progression of disease.

Our study has several limitations. First, this was a single-center, retrospective, cross-sectional study, and it is unable to provide a causal relationship between gut microbiota and NLR. Second, we only included a relatively young and healthy population, and data on subjects with disease are limited. Third, the clinical parameters such as symptoms or diagnosis were not acquired because we used health check-up materials, not medical records. Instead, data on medical, past, family, and medication history from the self-reported questionnaire were used. Some subjects tend to misreport or forget to include their history. However, this is the largest population-based study that demonstrated the association between the gut microbiome and NLR, showing differences in the diversity and abundance of bacteria between NLR groups.

This study investigated the association between the gut microbiota and NLR in a large healthy population. The diversity of the gut microbiota increased in subjects with a lower NLR. Furthermore, a higher NLR was associated with increased abundance of IBD-related gut microbiota and decreased abundance of beneficial bacteria. The findings of the present study highlight the role of NLR as a marker of intestinal inflammation because it reflects changes in the gut microbiota. Further long-term chronological studies are needed to determine the mechanism of the gut microbiota’s influence on NLR.

## Method

### Study population and design

A total of 1,463 Korean men and women between the ages of 25 and 78 years with a comprehensive annual or biennial physical examination between June 2014 and September 2014 at Kangbuk Samsung Hospital Healthcare Screening Center in the Republic of Korea were initially screened (Fig. 1)^49^. Among them, subjects who met the following criteria were excluded: 1) Subjects who received antibiotics within six weeks of enrollment or lipid-lowering drugs or probiotics within four weeks of enrollment due to the medication’s influence on gut microbiota (n = 141); 2) Subjects who did not have NLR data obtained during the physical examination (n = 5); and 3) Subjects whose fecal samples contained < 1,000 sequences per sample (n = 8). Finally, 1,309 subjects were included for analysis.

Demographics, laboratory data, past medical history, and other clinical data were obtained from the medical record, and questionnaires at the time of physical examination. This study protocol was approved by the Institutional Review Board of Kangbuk Samsung Hospital (KBSMC 2013-01-245-12). After explaining the nature and possible consequences of the study, we obtained written informed consent from all study participants. All applicable institutional and governmental regulations concerning the ethical use of human volunteers were followed during this research.

### DNA extraction from fecal samples and bacterial 16S rRNA gene sequencing

Fecal samples were frozen at −20 °C immediately after collection and stored at −70 °C within 24 hours. Within one month of receiving the samples, the MO Bio PowerSoil® DNA Isolation Kit (MO BIO Laboratories, Carlsbad, CA, USA) was used for DNA extraction from the fecal samples according to the manufacturer’s instructions. For amplification and sequencing of the DNA to analyze the bacterial communities, the methods described in a previous study were used^49^. Amplified genomic DNA was obtained using fusion primers targeting the variable V3 and V4 regions of the 16 S rRNA genes with indexing barcodes. The Illumina Miseq platform (Illumina, San Diego, CA, USA) was pooled for sequencing all samples according to the manufacturer’s specifications^50,51^.

### 16S rRNA gene compositional analysis

The DADA2 pipeline of the QIIME2 package (https://qiime2.org)^52^ was performed to generate unique sequence variants by filtering low quality samples and chimera. Since unique grouping sequences produce the “operational taxonomic units (OTUs)” from DADA2, they are regarded as 100% OTU and are typically referred to as sequence variants. The Feature Table was generated from the QIIME2 software, and it was the equivalent of the biom table and the representational sequence file. Almost identical (99%) sequence which was homology to an optimized version of the gene from the GreenGenes database (version 13.8) containing the V3-V4 region to detect taxonomies, mapped the sequences.

### Statistical analysis

The values of NLR were transformed using the natural logarithm (ln) to construct a normal distribution. After transformation, we divided the subjects into three groups according to NLR quartile (Q): lower Q (the lowest 25% of the NLR range), middle 2Q (from the next lowest 25% of the NLR range to the second highest 25% of the NLR range), and upper Q (the highest 25% of the NLR range). We also compared the lower Q to the higher 3Q, which included the middle 2Q and upper Q groups (the highest 75% of the NLR range) to investigate various aspects of the microbiota. All basic statistical analyses were performed with SPSS version 24.0 (SPSS Inc., Chicago, IL, USA). QIIME2 (version 2018.04) was utilized for exploratory and differential microbial composition analyses^52^. Alpha diversity measures of richness, community diversity, evenness, and phylogenetic diversity of gut microbial taxa were presented as observed OTU, Shannon index^53^, Pielou’s evenness^54^, and Faith’s phylogenetic diversity (PD)^55^, respectively. For measuring beta diversity, unweighted and weighted UniFrac^56^ values were calculated to determine the dissimilarity between groups. Diversity between the NLR groups was compared using pairwise PERMANOVA^57^. A *P-value* < 0.05 was considered statistically significant.

Correlation and comparison between the abundance of taxa and NLR were calculated using the Multivariate Association with Linear Models (MaAsLin) software package (https://huttenhower.sph.harvard.edu/maaslin)^58^ of RStudio (version 0.98.983). Confounding variables (age, sex, smoking status, and body mass index [BMI])-adjusted coefficients (CE) were estimated using MaAslin. All analyses of MaAslin were conducted using the default options. The FDR (Benjamini-Hochberg) method was used to adjust multiple comparisons on each phylogenetic level. A *q-value* < 0.05 was considered statistically significant.

## Electronic supplementary material

Supplementary Information

## Data Availability

All the supporting data is provided as supplementary files.
