# Supplementary material for: Association between Neutrophil-to-Lymphocyte Ratio and Gut Microbiota in a Large Population: a Retrospective Cross-Sectional Study
Source: Sci Rep. 2018 Oct 30;8:16031. doi: 10.1038/s41598-018-34398-4 (PMC6207698; doi:10.1038/s41598-018-34398-4)
Supplement: Supplementary file 1 — Supplementary Information [file 41598_2018_34398_MOESM1_ESM.pdf]

Association between Neutrophil-to-Lymphocyte Ratio and Gut Microbiota in a Large Population: a Retrospective Cross-Sectional Study

Hee-Young Yoon[1], Han-Na Kim[2], Su Hwan Lee[1], Soo Jung Kim[1], Yoosoo Chang[3,4], Seungho Ryu[3,4], Hocheol Shin[5], Hyung-Lae Kim[6], Jin Hwa Lee[1]

[1]Division of Pulmonary and Critical Care Medicine, Department of Internal Medicine, College of Medicine, Ewha Womans University, Seoul, Republic of Korea

[2]Medical Research Institute, Kangbuk Samsung Hospital, Sungkyunkwan University, School of Medicine, Seoul, South Korea.

[3]Center for Cohort Studies, Total Healthcare Center, Kangbuk Samsung Hospital, Sungkyunkwan University, School of Medicine, Seoul, South Korea.

[4]Department of Occupational and Environmental Medicine, Kangbuk Samsung Hospital, Sungkyunkwan University, School of Medicine, Seoul, South Korea.

[5]Department of Family Medicine, Kangbuk Samsung Hospital, Sungkyunkwan University School of Medicine, Seoul, South Korea.

[6]Department of Biochemistry, College of Medicine, Ewha Womans University, Seoul, Republic of Korea

Table S1. Commodities between neutrophil-to-lymphocyte-ratio groups based on quantile distribution.

Variables	Lower Q	Middle 2Q	Upper Q	P-value
No	328	653	328	
Dyslipidemia	61 (18.7)	94 (14.4)	48 (14.6)	0.194
Liver disease*	50 (15.3)	89 (13.6)	89 (10.1)	0.123
Colon polyp	44 (13.5)	93 (14.2)	36 (11.0)	0.359
Thyroid disease†	42 (12.8)	91 (13.9)	42 (12.8)	0.840
Hypertension	34 (10.4)	72 (11.0)	42 (12.8)	0.590
GB disease	15 (4.6)	34 (5.2)	14 (4.3)	0.791
History of tuberculosis	14 (4.3)	22 (3.4)	10 (3.0)	0.665
Osteoarthritis	12 (3.7)	25 (3.8)	6 (1.8)	0.229
History of malignancy‡	9 (2.8)	22 (3.4)	15 (4.6)	0.430
Benign prostate hypertrophy	9 (2.8)	26 (4.0)	8 (2.4)	0.363
Osteoporosis	6 (1.8)	10 (1.5)	1 (0.3)	0.170
Chronic obstructive pulmonary disease	3 (0.9)	6 (0.9)	2 (0.6)	0.869
Stroke	3 (0.9)	5 (0.8)	2 (0.6)	0.903
Heart disease	2 (0.6)	5 (0.8)	1 (0.3)	0.683
Coronary disease	2 (0.6)	5 (0.8)	1 (0.3)	0.683

Data are presented as number (%).

NLR, neutrophil-lymphocyte-ratio; Lower Q, < 25% of NLR range; Middle 2Q, ≥ 25% to < 75% of NLR range; Upper Q ≥ 75% of NLR range

*Liver disease included hepatitis B, hepatitis C, liver cirrhosis, and fatty liver

†Thyroid disease included

‡Malignancy included breast, thyroid, stomach, liver, lung, colon, cervix, and prostate cancer

Table S2. Nutritional status between neutrophil-to-lymphocyte-ratio groups based on quantile distribution.

Variables	Lower Q	Middle 2Q	Upper Q	P-value
No	328	653	328	
Total energy, kcal/day	1406.5 (596.1)	1425.2 (659.5)	1389.9 (649.2)	0.776
Total protein, g/day	49.6 (24.6)	48.5 (25.1)	47.6 (24.7)	0.683
Total fat, g/day	28.3 (18.4)	28.1 (19.5)	28.2 (19.0)	0.989
Total carbohydrate, g/day	235.0 (100.2)	241.0 (113.3)	233.1 (112.6)	0.605
Total calcium mg/day	308.7 (198.8)	307.8 (203.5)	321.2 (228.5)	0.701
Total phosphorus, mg/day	720.7 (338.7)	710.0 (346.6)	706.4 (348.9)	0.888
Total vitamin	353.3 (262.0)	336.9 (259.5)	326.4 (235.1)	0.495
Total sodium, mg/day	1663.5 (1073.8)	1609.0 (1061.2)	1653.8 (999.5)	0.756
Vitamin B1, mg/day	0.85 (0.46)	0.84 (0.46)	0.80 (0.42)	0.420
Vitamin C, mg/day	76.1 (59.0)	71.5 (68.9)	68.7 (56.3)	0.425
Folate, mg/day	155.9 (99.3)	151.1 (101.9)	149.9 (99.9)	0.776
Retinol, ug/day	71.2 (60.0)	68.7 (61.3)	74.6 (63.2)	0.481
Fiber, g/day	3.9 (2.4)	3.8 (2.5)	3.7 (2.1)	0.592
Cholesterol, mg/day	171.8 (137.1)	166.2 (137.4)	174.7 (136.0)	0.710

Data are presented as mean (standard deviation) or number (%).

NLR, neutrophil-lymphocyte-ratio; Lower Q, < 25% of NLR range; Middle 2Q, ≥ 25% to < 75% of NLR range; Upper Q ≥ 75% of NLR range

Table S3. Baseline characteristics between the lower quartile and higher 3 quartile neutrophil-to-lymphocyte ratio quartile groups

Variables	Lower Q	Higher 3Q	*P-value*
No	328	981	
Age	46.4 (8.9)	45.4 (8.9)	0.074
Male	214 (65.2)	598 (61.0)	0.166
Body mass index, cm/kg^2	23.7 (2.9)	23.6 (3.2)	0.651
Smoking status			0.143
Never	168 (56.0)	552 (56.3)	
Former	69 (23.0)	216 (22.0)	
Current	63 (21.0)	154 (15.7)	
Smoking amount, pyrs	6.0 (9.7)	6.0 (10.6)	0.990
NLR	1.1 (0.2)	1.9 (0.7)	<0.001
White blood cell, 10^3/mm^3	5.3 (1.3)	6.0 (1.5)	<0.001
Neutrophil, %	45.1 (4.6)	45.1 (4.6)	<0.001
Lymphocyte, %	44.7 (4.4)	32.3 (5.3)	<0.001
Hemoglobin, g/dL	14.3 (1.4)	14.3 (1.5)	0.646
Platelet, 10^3/mm^3	243.5 (51.8)	248.7 (60.0)	0.157
C-reactive protein, mg/dL	0.09 (0.19)	0.11 (0.17)	0.222

Data are presented as mean (standard deviation) or number (%).

NLR, neutrophil-lymphocyte-ratio; Lower Q, < 25% of NLR range; Higher 3Q, ≥ 25% of NLR range

Figure S1. Box plots of alpha-diversity indices comparing the lower quartile and the higher 3 quartile neutrophil-to-lymphocyte ratio groups

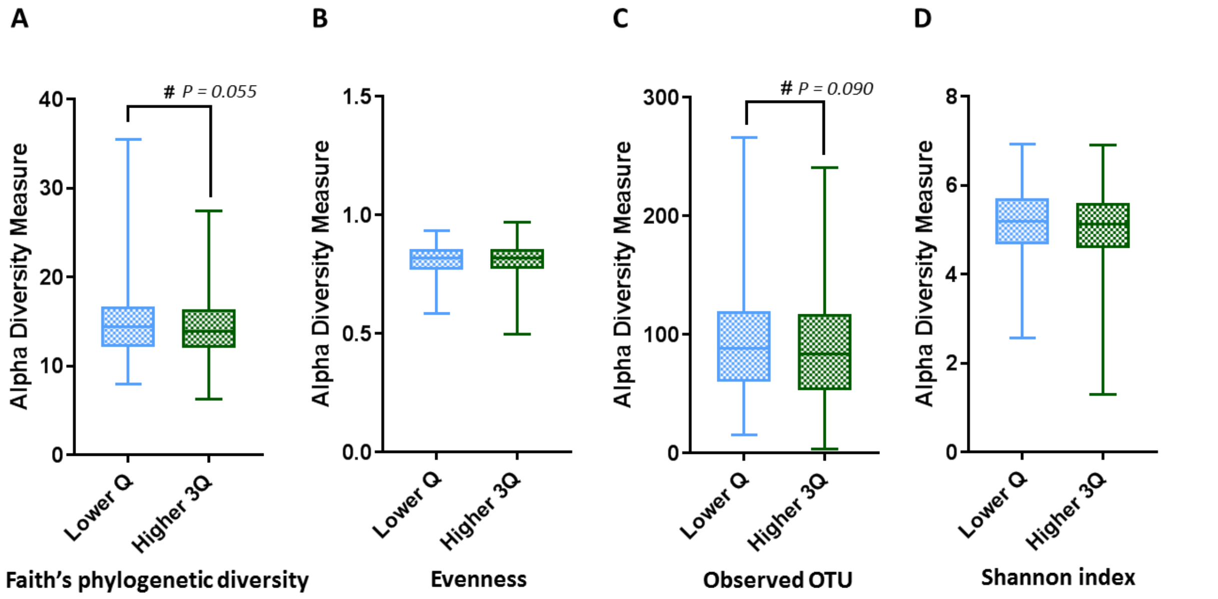

(A) Faith’s phylogenetic diversity (B) Pielou evenness (C) Observed operational taxonomic unit (D) Shannon index

OTU, operational taxonomic unit; Lower Q, < 25% of NLR range; Higher 3Q, ≥ 25% of NLR range

**P* <.05; #*P*<.0.1

Figure S2. Comparison distance of Unweighted UniFrac beta-diversity from the lower quartile group to the higher 3 quartile neutrophil-to-lymphocyte ratio group.

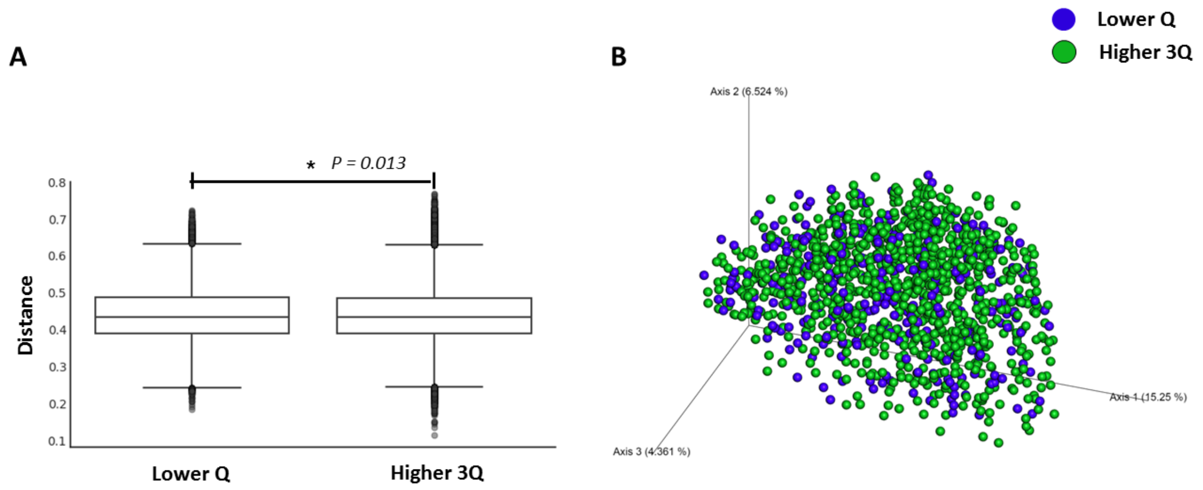

(A) Distance from the lower group (B) Principal coordinates analysis plot

Lower Q, < 25% of NLR range; Higher 3Q, ≥ 25% of NLR range

**P* <.05

Figure S3. Comparison of Relative abundance between the lower quartile and higher 3 quartile neutrophil-to-lymphocyte ratio groups

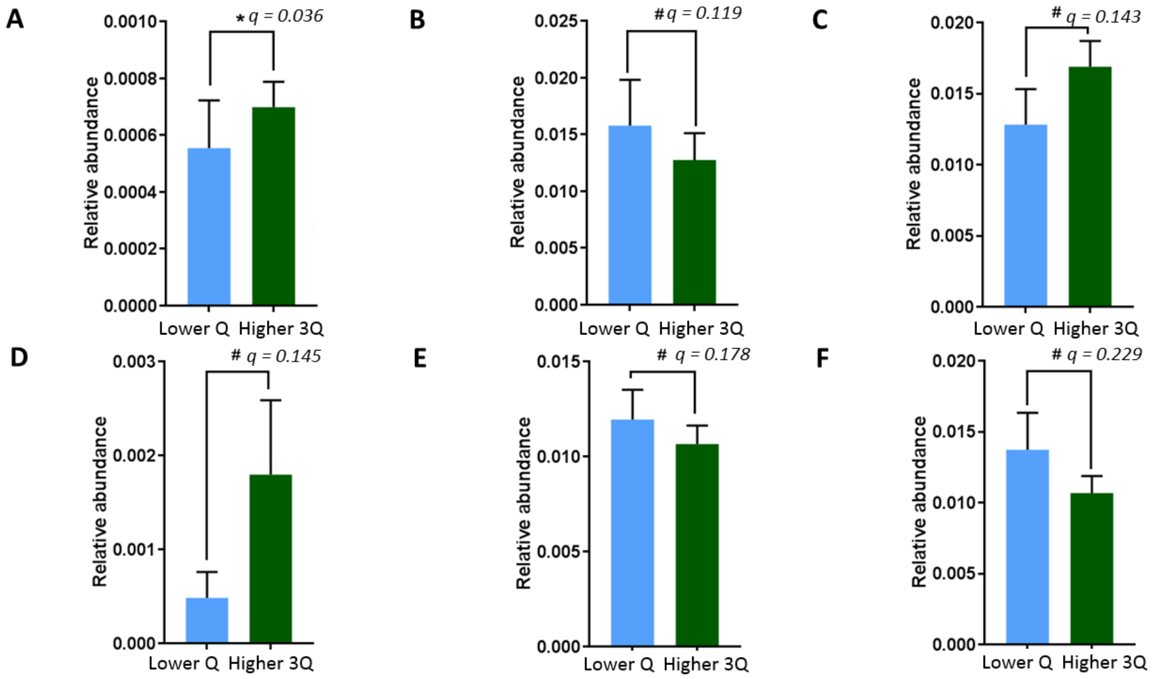

(A) genus *Bilophila* (B) *Prevotella stercorea* (C) genus *Dialister* (D) *Bacteroides eggerthii* (E) genus *Phascolarctobacterium* (F) genus *Lachnospira*

Lower Q, < 25% of NLR range; Higher 3Q, ≥ 25% of NLR range

*q <.05; #q<.0.25
